# Diet Quality and Level of Nutrition Knowledge among Young People with Orthorexic Tendencies

**DOI:** 10.3390/nu14204333

**Published:** 2022-10-17

**Authors:** Natalia Kaźmierczak-Wojtaś, Mariola Drozd

**Affiliations:** 1 Department of Psychology, Medical University of Lublin, 20-059 Lublin, Poland; 2 Department of Humanities and Social Medicine, Medical University of Lublin, 20-059 Lublin, Poland

**Keywords:** orthorexia, nutrition knowledge, diet quality

## Abstract

The aim of the study was to determine the level of nutrition knowledge and diet quality, understood in terms of healthy and unhealthy eating habits, among young people with orthorexic tendencies. The participants were school students, university students, and those employed in the Lublin region (N = 473). The data were collected by means of a questionnaire. The participants were asked to provide socio-demographic data through filling in the ORTO-15 questionnaire and the Dietary Habits and Nutrition Beliefs Questionnaire (KomPAN). The participants obtained results ranging from 9.3 to 100 (M = 31.15; SD = 11.81) in the non-healthy diet index, from 0.4 to 78.6 in the pro-healthy diet index (M = 21.79; SD = 11.08), and from 0 to 23 in the domain of nutrition knowledge (M = 13; SD = 4.23). A variance analysis showed no significant differences between the pro-healthy diet index and the level of nutrition knowledge. The group with orthorexia obtained statistically higher results in the pro-healthy diet index. Those with a tendency toward orthorexia obtained statistically higher results in the non-healthy diet index. The variance analysis showed that the level of nutrition knowledge of those not focused on healthy foods was significantly lower than in the other groups. The results of the ORTO-15 questionnaire correlated negatively with the pro-healthy diet index and the level of nutrition knowledge, and positively with the non-healthy diet index. We concluded that: 1. the orthorexic group and the group with a tendency toward orthorexia could be characterized with a moderate intensity of a healthy diet and a low intensity of a non-healthy diet; and 2. the level of nutrition knowledge in the orthorexic group did not significantly differ from that of the other groups.

## 1. Introduction

Orthorexia nervosa (ON, from Greek: *ortho* meaning “right”/“correct” and *orexis* meaning “appetite”/“desire”) is defined as “a pathological fixation with healthy food”. The term was introduced by S. Bratman in 1997 in *Yoga Journal* to indicate the existence of novel, unhealthy eating habits [1]. Orthorexic behavior manifests itself through rigorous dieting and the avoidance of foods containing preservatives, dyes, flavors, pesticides, or genetically modified foods [2]. Those suffering from orthorexia limit or eliminate specific types of foods from their diet, e.g., meat, dairy products, cereals, ready-prepared products, non-seasonal products, or products rich in fat, salt, or sugar [3,4]. A common practice is also a strict reliance on organic foods [5,6]. A list of acceptable products may vary from person to person; however, what is typical of the group is the progressive character of the imposed food restrictions. The preparation of meals is also kept under strict control as well as the diet well in advance and the selection of products regarded as healthy and rich in nutrients. Everyday activities are driven by eating-oriented thoughts and behavior [7]. Any violation of the self-imposed eating rules may result in a want to discipline oneself, either through the intensification of food restrictions or through fasting, negatively impacting one’s self-esteem. Furthermore, any deviations from the diet lead to a strong feeling of guilt and shame [6,8,9]. A total fixation on healthy eating results in neglecting personal goals and values, school assignments, professional duties, or interpersonal relationships. Those with ON avoid eating out and do not want to get involved in interactions with people who do not share their beliefs, which may result in social isolation and may have a negative effect on their wellbeing [2,9,10,11,12]. A few may feel morally superior to those who do not follow such a diet and eat “unhealthy” foods [8,9,10]. What becomes of essence for those suffering from ON is the highest quality of food they consume [9,12,13,14,15,16]. Orthorexia can be suspected when the described behavior does not ease and has a negative influence on the wellbeing of an individual [8].

Orthorexia has no official definition. It has not been included in the International Statistical Classification of Diseases and Health Problems (ICD-11) or in the American Psychiatric Association’s classification of mental disorders (DSM-5); the diagnostic criteria cited in the literature remain a proposal [13] The challenge with the classification and diagnosis of ON arises, among others, from its similarity to other nosological units. The literature on the subject emphasizes that ON has features in common with anorexia nervosa (AN) and obsessive-compulsive disorder (OCD) [2,17,18].

Both ON and AN are characterized by a tendency toward perfectionism, high coexisting anxiety, and the need to control [2,3,9,19,20]. In both cases, there is also an excessive focus on eating [3,6,13]; however, a few differences in this respect can be observed. People with ON focus on the quality and purity of food whereas people with AN primarily focus on the amount of food consumed [8,11,21]. Several scientists believe that fixations on food quality or type are also present in AN because people with this disorder also put in place certain rules about their diet [22,23,24]. Therefore, focusing on the quality and type of food consumed may not be a characteristic feature of ON. The rigid selection and gradual reduction of “allowed” products is present in both disorders; people with ON impose dietary restrictions to achieve optimal health and not because of a fear of obesity, as is the case with AN [8,14,25,26]. Deviations from self-imposed nutritional rules are identified by both groups as a lack of self-control [8]. In addition, symptoms are perceived as egosyntonic in both disorders, which may contribute to a low motivation for treatment [3]. Until now, it was believed that significant and deliberate weight loss and a distorted perception of one’s own body comprised the clinical picture of AN; these features do not occur in the course of ON [3,8,27,28]. Nevertheless, recent studies have shown that there is a link between ON and the desire to lose weight, inadequate assessments, and lower body acceptance [26,29], which indicates the close relationship between ON and eating disorders (EDs), especially AN. It is worth noting that the similarities and differences between the discussed disorders have not been empirically established [30] and, therefore, require further study.

Several researchers believe that ON and AN should be treated as a continuum of the same psychopathological dimension with different degrees of nuisance [12,31]. Mac Evilly [32] suggested that ON should be considered to be a risk factor or an initial stage of ED development, rather than classifying it as a separate disease entity. The eating behavior observed in the course of ON may become more restrictive and compulsive over time and, consequently, lead to a full-blown ED [33].

Bratman (2017) believes that one can distinguish two stages in the course of ON; so-called “healthy orthorexia”, where the individual is interested in healthy eating and not showing pathological features, and “orthorexia nervosa”, where the individual is obsessed with a healthy diet, referred to at this stage as a pathology.

Other authors believe that ON may be a comorbid disorder or may serve as a coping strategy for an ED [16,26,34]. Focusing on healthy foods and a lack of interest in low-calorie foods can help increase the variety of foods eaten and reduce the risk of weight loss. Patients remain highly selective in their choices and maintain control over their eating behavior, but at the same time begin to eat more food. Thus, this could be the first step to recovery from an ED [34].

ON, in addition to being similar to an ED, may also coincide with OCD or be a subtype of OCD [11,35,36]. Common symptoms include persistent obsessions (e.g., thinking about healthy food and meticulous meal planning), repetitive activities (e.g., ritual meal preparation, weighing products, and checking labels)) [18], social dysfunction, and a low quality of life as a consequence of obsessive behavior [7,37]. Despite a few similarities, the symptoms of OCD are egodystonic whereas in the case of ON, they are egosyntonic [8,9,37].

The proposal to include ON among the currently valid psychiatric classifications has not been widely accepted. Undoubtedly, further studies are needed to refine the clinical picture of ON, which may help in the final decision about its classification.

Rational eating habits are a prerequisite of a healthy lifestyle and play a substantial role in the appropriate growth and development of individuals. Rational eating habits mean sensible, proper nutrition; i.e., introducing ingredients from all groups of the food pyramid into the daily diet in the right amount as well as the right amount and frequency of meals. A rational diet is also a well-balanced diet [36]. Unhealthy eating habits are the opposite of rational, healthy eating habits. Unhealthy eating habits are two-fold. On the one hand, they are associated with nutritional deficiencies in the diet; on the other hand, with an inadequate quantity and frequency of meals consumed [36]. Unhealthy eating habits are a direct cause of or a risk factor behind a number of medical conditions. One of the indicators of healthy eating habits is the level of nutrition knowledge. Those who are aware of the importance of a balanced diet for the prevention of chronic metabolic disorders are more prone to adopt a healthy lifestyle [38]. The level of nutrition knowledge and rational eating habits shape a healthy lifestyle and thus have an impact on health. In this study, we attempted to measure the level of nutrition knowledge and to assess diet quality in terms of healthy and unhealthy eating habits exhibited by young people. We put forward the following hypotheses:

**H1:**  *People with a higher risk of orthorectic behavior are characterized by a higher level of nutrition knowledge;*

**H2:**  *The diet of people with a higher risk of orthorectic behavior is characterized by a higher intensity of healthy features and a lower intensity of unhealthy features.*

## 2. Materials and Methods

The study was carried out in 2019 among young people; i.e., school students, university students, and those employed in the Lublin region in Poland. The study was conducted in 3 secondary schools (2 high schools and 1 technical high school; 5 grades altogether) and 3 universities (Medical University of Lublin, Catholic University of Lublin, and the University of Economics and Innovation in Lublin). The schools and universities were selected at random after taking into account all entities located in the Lubelskie Voivodeship. Afterwards, for each school, the classes and groups of students were also randomly selected. To ensure a high return of completed questionnaires, the researchers selected classes and groups of students that could be reached directly (during classes). Teachers and other staff members aged up to 35 present on the day of the study were also asked to participate. In total, the questionnaire was distributed to 600 respondents. The participation rate was 89.7%. After an initial verification, 473 complete and correctly filled-in questionnaires qualified for a further analysis.

The data were obtained through a traditional diagnostic method; i.e., a pencil–paper questionnaire. Upon obtaining oral consent to take part in the study, the participants were informed of the aim and anonymity of the study and were instructed on how to complete the questionnaire. The inclusion criteria were age (i.e., young adults aged 16–35), informed consent to participate, and no symptoms of eating disorders (confirmed by the EAT-26 questionnaire). The respondents were asked to complete the questionnaire during classes/lectures. The survey questionnaire consisted of three tools: the ORTO-15 questionnaire; the KomPAN questionnaire; and the Eating Attitudes Test-26 (EAT-26). First, the participants provided their socio-demographic data (e.g., sex, age, height, BMI, education, and place of living), and then filled in the ORTO-15, KomPAN, and EAT-26 questionnaires. The obtained data were transferred to MS Office Excel and then subjected to a statistical analysis. A Bioethical Committee Agreement from the Medical University of Lublin was obtained for the study (No: KE0254/234/2016).

### 2.1. ORTO-15 Questionnaire 

This questionnaire consists of 15 items describing the intensity of orthorexic behavior, referring to the cognitive and emotional aspects connected with eating as well as the clinical symptoms of ON. The respondents provide answers on a four-level Likert scale (always, often, sometimes, and never). The responses were matched with point values from 1 to 4. Several of the responses were re-coded (their values were reversed) in accordance with the answer key. The responses indicating ON were of value 1 whereas those indicating healthy eating habits were of value 4. The authors established a cut-off value at the level of 40 points. A range of 40–60 points indicated a proper eating behavior, with subjects not showing any symptoms of a disorder. Lower scores were interpreted as indicative of a tendency toward ON. The ORTO-15 questionnaire is the most frequently used tool to measure orthorectic behavior [11,19,39]. 

Due to a problem with the internal consistency of the ORTO-15 questionnaire, we aimed to verify the reliability of this tool. The ORTO-15 questionnaire was adapted to Polish conditions by Brytek-Matera et al. [40]. As a result of their analyses, the authors received a 9-item tool with a Cronbach’s alpha value of 0.644. The results indicated an acceptable reliability, but the Nunnally criterion (acceptable level of reliability threshold α > 0.70) was not met [41]. In connection with the above, we decided to re-check the reliability of the tool in question and, on the basis of the results, we removed certain items. As the calculated Cronbach’s alpha reliability coefficient for the original 15-item version of the ORTO-15 questionnaire was 0.259 and for the Polish version it was 0.651, a decision was made to remove the questionnaire items that were the least correlated with the overall result. After removing another 3 items, the reliability of the tool increased. As a result, a 6-item tool (items 4, 6, 10, 11, 12, and 14) was obtained ORTO-6. This study relied on a 6-item version of the questionnaire, with a Cronbach’s alpha value of 0.696. The result was satisfactory and acceptable. The final 6-item version was characterized by the highest reliability that was achieved, taking into account the different number of items in the ORTO-6 questionnaire.

The group was divided based on a statistical criterion derived from psychometry and an empirical criterion. The statistical criterion was used to divide the sample into extreme groups in order to assess the differences between them. The criterion required that 25–27% of the total number of cases was drawn from each end of the distribution of results (so-called “tails”). In order to maximize the proportion of the obtained intergroup difference to the SD, in this study a proportion of the extreme groups of 27% was used. The statistical criterion allowed us to show the internal differentiation of the group of respondents, but did not refer to the clinical picture; therefore, an additional empirical criterion was applied. This criterion was determined on the basis of the Polish validation of the ORTO-15 questionnaire [42]. We identified four ranges for the results:

1. 6–7 points - orthorectic behavior;
2. 8–11 points - tendency toward orthorectic behavior;
3. 12–15 points - appropriate eating habits;
4. 16–24 points - low food interest [43].

It should be highlighted that the ORTO-15 questionnaire is of a self-descriptive character and the proposed ranges did not offer a diagnosis per se; rather, they provided an insight into the progressive character of a behavior indicating the risk of ON. Hence “the group with orthorexia” described those participants who showed the greatest tendency toward orthorectic behavior.

### 2.2. The Dietary Habits and Nutrition Beliefs Questionnaire 

The questionnaire designed to study dietary habits and beliefs, known as KomPAN, is an updated and extended version of the Questionnaire of Eating Behaviour. The KomPAN questionnaire is made up of 4 parts, which provide information on dietary habits (A), the frequency of food consumption (B), nutrition beliefs (C), and lifestyle and personal data (D). To obtain a comprehensive evaluation of the quality of nutrition, we introduced two indexes on the basis of Part B; i.e., the one regarding foods that have a positive effect on health (“pro-healthy diet index”—pHDI-10 or Pro-Healthy Diet Index-10) and the other one regarding foods that have a negative effect on health (“non-healthy diet index”—nHDI-14 or Non-Healthy Diet Index-14). The respondents assessed the frequency of consuming particular products on a 6-level scale ranging from “never” to “several times a day”, which was then calculated into a daily frequency (times/day). The indexes were calculated by summing up the particular frequencies for 10 and 14 groups of products; these were then translated into an aggregate frequency of consumption expressed from 0 to 100 points [44] in line with the formulae:

- “pro-healthy diet index” (pHDI-10, in terms of points) = (100/20) × sum of the frequencies of consumption of 10 groups of foods (times/day);
- “non-healthy diet index” (nHDI-14, in terms of points) = (100/28) × sum of the frequencies of consumption of 14 groups of foods (times/day).

The higher the index value, the greater the intensity of favorable or unfavorable health conditions. The responses were coded in accordance with the guidelines provided by the authors of the questionnaire (1 point for each correct answer; 0 points for a wrong answer or an “unsure” answer). According to the recommendations of the authors of the questionnaire, on the basis of the obtained results we could distinguish between the groups of participants with a low (0–33 points), medium (34–66 points), or high (67–100 points) intensity of favorable or unfavorable health conditions. Part C of the KomPAN includes statements regarding food and nutrition. This allows for a differentiation between groups based on the level of nutrition knowledge; i.e., unsatisfactory (0–8 points), satisfactory (9–16 points), or good (17–25 points) [44]. In our study, both Part B and Part C of the questionnaire were subject to an analysis.

### 2.3. Data Analysis

The data obtained in the survey were subject to a statistical analysis using IBM SPSS Statistics software, version 23, which included:

- Descriptive statistics of the pro-healthy diet index and non-healthy diet index as well as the level of nutrition knowledge, calculated for the general group of participants and in line with a classification into four groups: those with a higher risk of orthorectic behavior (1); those with a tendency toward orthorectic behavior (2); those with appropriate eating habits (3); and those with a low food interest (4);
- A variance analysis with Tukey’s multiple comparison test, comparing four groups in terms of the pro-healthy diet index and non-healthy diet index and the level of nutrition knowledge;
- An analysis of the linear correlation (*r*—the Pearson’s coefficient) between the quantitative result of ORTO-6 and the pro-healthy diet index and non-healthy diet index and the level of nutrition knowledge.

For the tests used, we assumed a value of statistical significance of *p* < 0.05.

## 3. Results

A total of 473 participants took part in the study. The majority were women (70%, N = 331), with men constituting 30% of the group (N = 142). A high risk of orthorectic behavior was twice as likely among women (4.2%) than among men (2.1%). The mean age of the participants was 22.68 (SD = 4.58) and the mean BMI was 22.68 (SD = 3.61). The Chi2 test (Chi2 = 16.314; *p* = 0.001) showed a statistically significant relationship between gender and ON. Socio-demographic features such as age, place of residence, BMI, education, and profession did not have any impact on the results.

The detailed data are presented in Table 1.

The results of the pro-healthy diet index ranged from 9.3 to 100 (M = 31.15; SD = 11.81) whilst the results of the non-healthy diet index ranged from 0.4 to 78.6 (M = 21.79; SD = 11.08) (Table 2). Both the mean and median value pointed toward a low index of healthy and non-healthy diets. When it came to the level of nutrition knowledge, the results ranged from 0 to 23 (M = 13; SD = 4.23). Both the mean and median value pointed toward satisfactory nutrition knowledge.

The variance analysis showed that the four groups differed statistically in terms of the index of diet quality and the level of nutritional knowledge.

Those with a higher risk of orthorectic behavior obtained statistically higher results in the pro-healthy diet index than those with a tendency toward orthorectic behavior, those with appropriate eating habits, or those with a low food interest. Those with a low food interest obtained statistically lower results than those with a tendency toward orthorectic behavior and those with appropriate eating habits. Those with a tendency toward orthorectic behavior obtained statistically lower results in the non-healthy diet index than those with appropriate eating habits and those with a low food interest.

The variance analysis also demonstrated that the level of nutrition knowledge in the group with a low food interest was statistically lower than in the group with orthorectic behavior, with a tendency toward orthorectic behavior, or those with appropriate eating habits. The detailed data are presented in Table 3.

The results obtained from the ORTO-6 questionnaire correlated negatively with the pro-healthy diet index and the level of nutrition knowledge and positively with the non-healthy diet index. The lower the ORTO-6 results (a greater intensity of ON), the higher the nutrition knowledge and the greater the intensity of the pro-healthy diet and the lower the intensity of the non-healthy diet (Table 4).

## 4. Discussion

An analysis of diet quality can be performed on the basis of the frequency of the consumption of particular products. Such an analysis can be helpful in eliminating nutrition mistakes and in making appropriate nutrition choices. On this basis, there is a view that an evaluation of a diet can help prevent nutrition-based disorders. However, our study pointed toward a statistically higher value of the pro-healthy diet index in the group with orthorectic behavior than in the other groups. This meant that that group consumed products commonly regarded as healthy (i.e., wholemeal bread/bread rolls, wholegrain pasta, coarse-ground groats, fermented milk drinks, fish, legume-based foods, fruit, and vegetables) more often than the other groups. It could be assumed that these nutrition choices did not stem only from personal tastes but most of all from nutrition awareness. It was observed that the diet of the groups with a higher risk of orthorectic behavior and with a tendency toward orthorectic behavior could be characterized by a moderate intensity of a pro-healthy diet and a low intensity of a non-healthy diet in comparison with the other groups where the intensity of a pro-healthy diet and a non-healthy diet was low. The obtained results were consistent with hypothesis H2. A low intensity of pro-healthy and non-healthy eating habits in young people was supported by a study conducted by Galiński et al. [45]. It should be stressed that the non-healthy diet index was low in all the four groups, which could be viewed as a positive trend, with the lowest results obtained by the groups with a higher risk of orthorectic behavior and a tendency toward orthorectic behavior. This indicated that the respondents curbed their intake of unhealthy foods; i.e., white bread and bakery products, fast food, fried foods, meat or flour-based foods, cheese, sweets, tinned (jar) meats, sweetened carbonated or still drinks, energy drinks, or alcoholic beverages. A previously published study by one of the authors of this research also indicated a low consumption of unhealthy products in a group with a higher risk of orthorectic behavior [46].

It was concluded that the groups with a higher risk of orthorectic behavior and with a tendency toward orthorectic behavior made healthier nutrition-related choices than the groups following a healthy diet and not focused on healthy food. However, it ought to be noted that ON involves a pathological fixation with healthy eating. Therefore, in the case of ON, the behavior, which is initially healthy to the organism, develops into a pathology with many negative medical consequences. Relatively scarce available literature relating to this research area indicates a need for further studies on ON to be carried out in the future.

Eating habits are an important factor shaping the quality of human life [47]. The level of nutrition knowledge and health-oriented beliefs can have a major impact on diet and pro-health behavior [48,49]. A few authors have claimed that a higher level of nutrition knowledge correlates with healthy eating [50,51]; others downplay the role of such a correlation [45,52]. In the context of the topic discussed, it was to be expected that people at a high risk of orthorectic behavior would have a high level of nutritional knowledge as the disorder usually begins with a desire to improve their health, which naturally requires an interest in the topic of nutrition [33]. Our study confirmed that the group with a higher risk of orthorectic behavior possessed a satisfactory level of nutrition knowledge; nevertheless, this was of a similar level across all the studied groups. Only in the group with a low food interest was the level of nutrition knowledge statistically lower; however, it was still within a satisfactory range. The results obtained in our study did not confirm the H1 hypothesis concerning a higher level of nutrition knowledge in the group with a higher risk of orthorectic behavior. This was supported by other studies; e.g., by Czarnewicz-Kamińska and Gronowska-Senger [53], which concluded that the patient groups suffering from anorexia nervosa and bulimia could be characterized by a higher, yet not statistically significant, level of nutrition knowledge.

The available literature points to a relatively higher prevalence of ON among students of health-related fields (e.g., nutrition, medicine, and nursing [3,19,30,31,32,33,34,35,36,37,38,39,40,41,42,43,44,45,46,47,48,48,49,50,51,52,53,54,55]) or among representatives of medical professions (mostly dieticians and doctors [5,11,19,56,57]). Several studies note that due to the profile of their education, such students possess a greater knowledge relating to health, nutrition-related disorders, the rules of healthy dieting, and the consequences of not following these rules, which may be a potential risk factor of ON. On the other hand, other researchers have shown an inverse relationship between nutritional knowledge and the risk of ON; i.e., with an increasing knowledge of nutrition, the risk of ON tends to decrease [31,48,58,59]. Thus, a greater nutritional knowledge has the potential to influence the choice of healthy foods as well as an appropriate eating behavior. However, other studies point out that a growing awareness of the correlation between health and diet may not be correlated with the profile of education [54,60]. Our study did not confirm such a correlation, which may result from the specifics of the studied group

## 5. Limitations and Strengths of the Study

The correlation between ON and the diet quality and level of nutrition knowledge has not been extensively studied so far. Our study attempted to bridge this gap and the obtained results should be treated as a complementation of the data on ON as emerging from the available studies. A limitation of our study was the recruitment of the respondents among school students and university students who filled in the questionnaires in class, which limited their anonymity. The available literature suggests that a feeling of complete anonymity can increase the accuracy of given answers, especially if they are of a delicate character [61]. A few of the participants, whilst filling in the questionnaires in a big group, could provide expected answers even if they could not be verified. The study involved mainly young people (aged 16–35) because such a group is mostly subject to orthorexic behavior [62,63,64,65]. The respondents were mostly women, which did not make it a representative group in the population of young people. A study in an extended group should be conducted in the future. We relied on the ORTO-15 questionnaire, whose value has been questioned recently due to its varying internal integrity (Cronbach’s alpha varying from 0.14 to 0.82 with a mean value of Cronbach’s alpha of 0.55), its low reliability, or doubts concerning its cut-off point [66,67,68]. Nevertheless, at the time of our study, it was the only available ON diagnostic tool in Polish, so no other choices could have been made. Despite its limitations, ORTO-15 is a common tool that allows researchers to compare the obtained results. As there are only a few studies dealing with nutrition-related practices in the course of ON [46,69,70], further studies on ON should focus on developing a reliable tool to evaluate orthorexic behavior and to verify the obtained results.

## 6. Conclusions

The obtained results led to the following conclusions.

Those suffering from orthorexia or with a tendency toward orthorexia demonstrated a moderate intensity of a pro-healthy diet and a low intensity of a non-healthy diet.

The level of nutrition knowledge in the group with orthorexia was average and did not differ statistically from the other groups.

The level of nutrition knowledge and nutrition-related behavior indicated a few non-healthy tendencies in the studied group. This requires preventive educational measures to be taken aimed at raising awareness of the role of healthy eating. It should be noted that eating habits are formed early in human lives and solidify in adulthood and can be difficult to modify. It is particularly vital to shape appropriate attitudes and behavior toward pro-healthy dieting among young people. Special programs should be developed to promote the principles of healthy nutrition.

Aspects of the behavior observed in the group with a higher risk of orthorectic behavior were shared by the group with a tendency toward orthorectic behavior, which testified the need to distinguish a high-risk group and include it in future ON diagnostic tools.

The obtained results should act as a foundation for further studies on ON symptoms and may be helpful in understanding the specificity of ON. It is recommended to validate the study with a more representative group and the use of more reliable tools to measure ON.

## Figures and Tables

**Table 1:** Characteristics of the study group.

Variables	ORTO						Statistics
		Orthorexic Behavior	Tendency Toward Orthorexia	Appropriate Eating Habits	Low Food Interest	All	
Gender							Chi2
Female	N	14	101	157	59	331	Chi2 = 16.314 *p* = 0.001
	%	4.2	30.5	47.4	17.8	70.0	
Male	N	3	37	53	49	142	
	%	2.1	26.1	37.3	34.5	30.0	
Age							ANOVA
	Min	18.00	16.00	16.00	16.00	16.00	F = 0.178 *p* = 0.911
	Max	35.00	35.00	35.00	35.00	35.00	
	M	22.82	22.53	22.84	22.54	22.68	
	Me	22.00	22.00	22.00	21.00	22.00	
	SD	4.20	4.23	4.84	4.59	4.58	
Growth (cm)	Min	151.00	145.00	150.00	151.00	145.00	F = 2.324 *p* = 0.074
	Max	190.00	190.00	196.00	194.00	196.00	
	M	169.76	170.33	169.64	172.42	170.48	
	Me	170.00	169.00	169.50	172.50	170.00	
	SD	8.92	9.31	8.49	9.54	9.03	
Body weight	Min	42.00	41.00	40.00	38.00	38.00	F = 2.017 *p* = 0.111
	Max	88.00	125.00	130.00	117.00	130.00	
	M	62.12	66.14	65.54	69.06	66.39	
	Me	62.00	65.00	63.00	68.00	65.00	
	SD	12.64	13.50	14.15	15.77	14.35	
BMI	Min	16.56	16.69	16.02	16.67	16.02	F = 1.050 *p* = 0.370
	Max	30.08	35.37	37.98	33.90	37.98	
	M	21.44	22.64	22.62	23.04	22.68	
	Me	20.70	22.19	22.10	22.48	22.21	
	SD	3.53	3.28	3.66	3.91	3.61	
Place of residence:							Chi2
Village	N	7	61	95	47	210	Chi2 = 7.342 *p* = 0.602
	%	3.3	29.0	45.2	22.4	44.4	
City up to 100,000 residents	N	5	38	53	29	125	
	%	4.0	30.4	42.4	23.2	26.4	
City 100,000–300,000 residents	N	3	15	14	6	38	
	%	7.9	39.5	36.8	15.8	8.0	
City with over 300,000 residents	N	2	24	48	26	100	
	%	2.0	24.0	48.0	26.0	21.1	
**Education**							Chi2 = 17.483 *p* = 0.132
Primary education	N	0	0	1	0	1	
	%	0.0	0.0	100	0.0	0.2	
Lower secondary education	N	0	12	12	12	36	
	%	0.0	33.3	33.3	33.3	7.6	
Vocational education	N	0	3	3	0	6	
	%	0.0	50.0	50.0	0.0	1.3	
Secondary education	N	9	62	117	68	257	
	%	3.5	24.2	45.7	26.6	54.2	
Higher education	N	8	60	77	28	173	
	%	4.6	34.7	44.5	16.2	36.7	
Medical profile	N	3	23	32	8	66	
	%	37.5	38.3	41.5	28.6	38.1	
Humanistic profile	N	4	28	30	9	71	
	%	50	46.7	39.0	32.1	41.0	
Technical profile	N	1	9	15	11	36	
	%	12.5	15.0	19.5	39.3	20.9	

Min: minimum; Max: maximum; M: average; Me: median; SD: standard deviation; N: number; ANOVA: analysis of variance; F: Fisher test; *p*: statistical significance; Chi2: test.

**Table 2:** Indexes of diet quality and the level of nutrition knowledge in the general group of participants.

Hypothesis	KomPAN	Min	Max	M	Me	SD	Range (In Points)		
							Intensity of Nutritional Characteristics		
							Low	Medium	High
H2	Pro-healthy diet index	9.30	100.00	31.15	29.80	11.81	0–33	34–66	67–100
	Non-healthy diet index	0.40	78.60	21.79	21.10	11.08	0–33	34–66	67–100
							**Level of Nutritional Knowledge**		
							Insufficient	Sufficient	Good
H1	Level of nutrition knowledge	0.00	23.00	13.00	13.00	4.23	0–8	9–16	17–25

Min: minimum; Max: maximum; M: average; Me: median; SD: standard deviation.

**Table 3:** Indexes of diet quality and the level of nutrition knowledge in the particular groups.

Hypothesis	KomPAN	ORTO										
		Orthorexic Behavior		Tendency Toward Orthorexy		Appropriate Eating Habits		Low Food Interest		ANOVA		
		M	SD	M	SD	M	SD	M	SD	F	*p*	I.D. (Tukey’s Test)
H2	Pro-healthy diet index	41.09	11.05	33.41	12.36	30.90	12.14	27.18	8.67	10.382	0.000	1/2; 1/3; 1/4; 2/4; 3/4
	Non-healthy diet index	18.61	11.98	18.55	10.10	22.57	11.67	24.92	9.84	7.981	0.000	2/3; 2/4
H1	Level of nutrition knowledge	14.88	3.28	13.62	4.31	13.21	4.20	11.48	3.95	7.223	0.000	1/4; 2/4; 3/4

M: average; SD: standard deviation; ANOVA: analysis of variance; F: F-test; *p*: statistical significance; I.D.: individual differences.

**Table 4:** Correlations between the intensity of orthorexia as measured by ORTO-6 questionnaire and the level of nutrition knowledge as measured by KomPAN questionnaire.

Hypothesis	KomPAN	ORTO	
		R	*p*
H1	Pro-healthy diet index	−0.219	0.000
	Non-healthy diet index	0.230	0.000
H2	Level of nutrition knowledge	−0.203	0.000

R: Pearson correlation; *p*: statistical significance.

## Data Availability

The data presented in this study are available from Natalia KaźmierczakWojtaś.
